# LAMB1 promotes proliferation and metastasis in nasopharyngeal carcinoma and shapes the immune-suppressive tumor microenvironment

**DOI:** 10.1016/j.bjorl.2024.101551

**Published:** 2025-01-27

**Authors:** Enzi Feng, Xinyu Yang, Jie Yang, Qianqian Qu, Xiaojiang Li

**Affiliations:** The Third Affiliated Hospital of Kunming Medical University, Department of Head and Neck Surgery, Kunming, China

**Keywords:** Nasopharyngeal carcinoma, LAMB1, Cancer-associated fibroblasts, Metastasis

## Abstract

**Objective:**

Laminin subunit Beta-1 (LAMB1), a component of the extracellular matrix, has been reported to be implicated in the development and progression of cancer. However, the role of LAMB1 in Nasopharyngeal Carcinoma (NPC) remains unknown.

**Methods:**

Three NPC datasets were utilized to identify LAMB1 as a targeted gene. The correlation between LAMB1 expression and clinical characteristics, prognosis was explored. KEGG and GO enrichment analyses were conducted to investigate LAMB1’s functions in NPC. The CIBERSORT, xCell, MCPCOUNTER, and EPIC methods were used to assess the Cancer-Associated Fibroblasts (CAFs) and immune cells infiltration. We predicted LAMB1’s effect on treatment using TIDE, CTRP, and CellMine databases. Finally, Western blot, CCK-8, Transwell, and Wound scratch were employed to validate LAMB1’s effect on NPC cells.

**Results:**

LAMB1 was highly expressed in NPC. High-expression LAMB1 was correlated with poorer progression-free survival and impeded the infiltration of CD4^+^ T-cells, CD8^+^ T-cells and dendritic cells. It also diminished the expression of HLA and suppressed T-cells stimulation. Differential expressed cytokines and involved pathways were divergent across different level of fibroblasts infiltration. At high level of fibroblasts, LAMB1 indirectly inhibited immune cells by remolding extracellular matrix. But at low level of fibroblasts, LAMB1 directly suppressed immune response. Tumors with high LAMB1 level had weak responses to immunotherapy. In vitro experiment, LAMB1 significantly suppressed HLA-1 and enhanced the proliferation, migration, and invasion capabilities of NPC cells.

**Conclusion:**

High expression of LAMB1 is significantly associated with an immune-suppressive tumor microenvironment in NPC. LAMB1 enhances the proliferation, migration and invasion of NPC cells. These findings suggest that LAMB1 may serve as a prognostic biomarker for predicting NPC progression and a potential therapeutic target to enhance the efficacy of existing immunotherapies.

**Level of evidence:**

3.

## Highlights

• NPC exhibits significantly elevated expression of LAMB1.
• LAMB1 plays immune-suppressive role in NPC.
• LAMB1 promotes NPC cells proliferation, invasion and migration.

## Introduction

Nasopharyngeal Carcinoma (NPC) is a highly aggressive malignancy originating from the epithelium of nasopharynx. Globally, the World Health Organization (WHO) reported 120,434 new cases and 73,482 related deaths in 2022. This malignancy is prevalent in Asia and Africa, which together accounted for 92.2% of all new NPC cases. Specifically, China had the highest incidence with 51,010, representing 50.9% of new cases in Asia.^1^ Due to the nasopharynx’s deep location, NPC symptoms are initially inconspicuous. By the time of diagnosis, most patients have progressed to advanced stage. Some individuals with advanced disease remain resistant to current treatment, leading to disease progression. Further research is imperative to elucidate the pathogenic and metastatic mechanisms of advanced NPC and to find appropriate therapeutic targets.

LAMB1 is the major non-collagenous component of Extracellular Matrix (ECM) and implicated in tumor metastasis as well as poor prognosis in several cancers. In hepatocellular carcinoma, LAMB1 facilitates tumor progression through the PDGF/La axis3.^2^ In gastric cancer, LAMB1 enhances cancer cell growth and motility through the ERK/c-Jun pathway.^3^ Colorectal cancer patients have been found to exhibit elevated level of LAMB1 in their serum,^4^ suggesting that LAMB1 could act as a potential serum marker. However, the specific function and mechanism of LAMB1 in NPC remain unclear. Additionally, Cancer-Associated Fibroblasts (CAFs) are one of the most abundant stromal cells in the Tumor Microenvironment (TME). Increasing evidence suggests that CAFs involved in the remodeling of ECM, promoting the proliferation, migration, and invasion of tumor cells and impairing immune system surveillance.^5^, ^6^, ^7^

In this study, we found that LAMB1 was highly expressed in NPC, predominantly in malignant cells and stromal cells according to the single-cell map. High level of CAFs were observed in the group of high-expression LAMB1. Consequently, we investigated the function of LAMB1 at both high and low infiltration levels of CAFs to elucidate its role in the development of NPC.

## Methods

### Data collection and processing

The mRNA expression datasets analyzed in this study were sourced from the Gene Expression Omnibus (GEO) database. The datasets comprise GSE12452 (n = 41), GSE13597 (n = 28), GSE103611 (n = 48), and GSE102349 (n = 113). GSE12452 utilized the GPL570 platform (Affymetrix Human Genome U133 Plus 2.0 Array), encompassing 31 NPC tissues and 10 normal nasopharyngeal tissues. GSE13597 was based on the GPL96 platform (Affymetrix Human Genome U133A Array) and included 25 NPC tissues and 3 normal nasopharyngeal tissues. GSE103611 was based on the GPL19251 platform (Affymetrix Human Gene 2.0 ST Array), including 24 locally advanced NPC tissues with distant metastasis and 24 locally advanced NPC tissues without distant metastasis. GSE102349 utilized the GPL11154 platform (Illumina HiSeq 2000), including 113 NPC tissues. Data processing was performed using R software (v. 4.3.1).

### Targeted gene selection

The ‘limma’ package (v. 3.58.1) facilitated the identification of Differentially Expressed Genes (DEGs) in three datasets: NPC samples versus normal samples (GSE12452, GSE13597); NPC with distant metastases versus those without (GSE103611). Criteria included log2-fold change ≥ 1 and *p*-value < 0.05. Subsequently, the DEGs from the three datasets underwent cross-analysis to identify gene intersections, with results depicted through Venn diagrams. Ultimately, we decided to focus on the gene-LAMB1.

### Bioinformatic analysis of LAMB1

We initially used single-cell RNA-seq from Tumor Immune Single-Cell Hub 2 (TISCH2) database (http://tisch.comp-genomics.org/) to explore where LAMB1 was expressed (NPC_GSE150430). The new NPC dataset GSE102349 enabled validation and functional analysis of the target gene. Based on the LAMB1 expression level, the 113 samples were categorized into high and low expression groups. The correlations between clinical factors ‒ including Progression-Free Survival (PFS), clinical stage, and Tumor Infiltrating Lymphocytes (TILs) ‒ and LAMB1 were explored.

The relative abundance of CAFs within the matrix was assessed using three methods: xCell package (v. 1.1.0), MCPCOUNTER package (v. 1.2.0), and EPIC package (v. 1.1.7). We further divided samples into groups characterized by high or low CAFs, subsequently categorizing these groups into high or low LAMB1 expression levels. To investigate the function of the target gene and related signaling pathways, we performed GO and KEGG analyses. Subsequently, we compared infiltrating immune cells using the xCell algorithm. Differentially expressed cytokines were analyzed using the “KEGG Cytokine-Cytokine Receptor Interaction” gene set, downloaded from Gene Set Enrichment Analysis (GSEA) (https://www.gsea-msigdb.org). The score of immune-related function were analyzed using “The 29 immune signatures represented by 29 different gene sets”.^8^

Responses to immunotherapy in different LAMB1 expression groups were predicted using the Tumor Immune Dysfunction and Exclusion (TIDE) score (http://tide.dfci.harvard.edu/). Additionally, this study explored the correlation between LAMB1 and immune checkpoints. Cell line expression data and drug sensitivity information were obtained from the Cancer Therapeutics Response Portal (CTRP) database (https://portals.broadinstitute.org/ctrp/) to construct the predictive model. Subsequently, this model was employed to predict the IC50 of drugs for the GSE102349 dataset. Furthermore, data on LAMB1 expression and drug sensitivity were acquired from the CellMiner database (https://discover.nci.nih.gov/cellminer/home.do). A Pearson analysis was conducted to identify the four most sensitive drugs.

### Cell culture

The NPC cell lines (CNE1, CNE2, 6-10B) and the normal Nasopharyngeal Epithelial cell line (NP69) were generously provided by Dr. Huang (Kunming Medical University). All the cell lines used in this study had been authenticated. NPC cell lines were cultured in RPMI-1640 medium with 10% fetal bovine serum and 1% penicillin/streptomycin, and NP69 was cultured in K-SFM medium. All cell lines were cultured at 37 °C with 5% CO_2_.

### Western blot

Cellular proteins were extracted by lysing cells using RIPA lysate supplemented with protein inhibitors. Proteins were then separated using a 7.5% PAGE gel and subsequently transferred onto Polyvinylidene Fluoride (PVDF) membranes. The PVDF membrane was incubated with anti-LAMB1 polyclonal antibody (1:1000, Proteintech, China), anti-HLA-1 polyclonal antibody (1:10000, Proteintech, China), anti-β-Tubulin polyclonal antibody (1:20000, Proteintech, China) and anti-GAPDH monoclonal antibody (1:50000, Proteintech, China), followed by an HRP-conjugated secondary antibody.

### siRNA and transfection

Three siRNAs targeting LAMB1 gene, and one negative control siRNA were designed and synthesized by General Biosystems Corporation (China). The sequence of si-LAMB1-1: CAGCAGAAGCGUUGUGAAATT, UUUCACAACGCUUCUGCUGTT; si-LAMB1-2: GGUGAUAUUUCGUGCUUUATT, UAAAGCACGAAAUAUCACCTT; si-LAMB1-3: CUGCAUACUUUGGGAGAUATT, UAUCUCCCAAAGUAUGCAGTT; negative control: UUCUCCGAACGUGUCACGUTT, ACGUGACACGUUCGGAGAATT. NPC cells were transfected with siRNAs using Lipofectamine RNAiMAX (Invitrogen, USA) according to the manufacturer’s protocol.

### CCK8 assay

Cell suspension was evenly seeded at a density of 5,000 cells per well in a 96-well plate. After observing cell adhesion, 10 μL of CCK-8 was added and thoroughly mixed at 0-, 24-, 48-, 72-, and 96-hours. Subsequently, cells were incubated at 37 °C for one hour. Absorbance measurements were then taken at 450 nm.

### Transwell invasion assay

A Transwell chamber was utilized for cell invasion assays. The bottom of the upper chamber was coated with Matrigel solution, diluted in sterile deionized water. 600 μL of medium containing 10% FBS was added in the lower chamber. NPC cells were collected and resuspended in serum-free medium. Subsequently, 100 μL of the cell suspension, containing 5 × 10^4^ cells, was added to the upper chamber. Cells were then incubated for 24 hours. Samples were fixed with 4% paraformaldehyde and stained with crystal violet. Images were captured in multiple fields of view at 10× magnification.

### Wound scratch assay

NPC cells were seeded in a 6-well plate. When reaching 90%‒95% confluence, a straight line was drawn across the cell layer using a 200 μL pipette tip. Wound healing was photographed at 0-, 24-, and 48-hours.

### Statistical analysis

Statistical analyses for the bioinformatics part of this study utilized R software (v. 4.3.1) and an online tool (https://www.xiantaozi.com/products). For in vitro experiments, data analysis was conducted with GraphPad Prism software (v. 9.0.0). Comparisons between two groups employed the unpaired *t*-test. Quantified band intensity differences among groups were analyzed with analysis of variance (ANOVA); *p*-value < 0.05 was considered statistically significant.

## Results

### Identification of DEGs in NPC

**Fig. 1:**
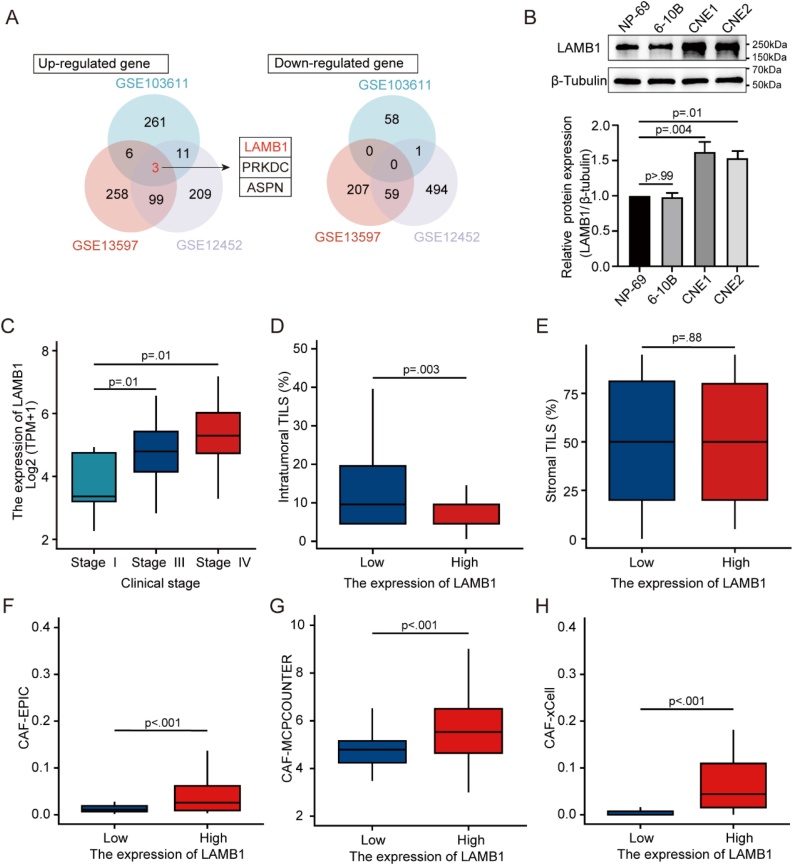
The expression of LAMB1 in NPC cells and the association between LAMB1 and clinical stage, TILs, CAFs. (A) The DEGs intersection of 3 datasets (GSE12452, GSE13597 and GSE103611) included 3 identical upregulated genes and 0 identical downregulated gene. (B) LAMB1 expression in normal Nasopharyngeal Epithelial cells (NP69) and NPC cells (6-10B, CNE1, CNE2). The association between LAMB1 expression and clinical stage (C), TILs (D-E) and CAFs (F-H) in GSE102349.

Analysis of DEGs in datasets GSE12452, GSE13597, and GSE103611 utilized the ‘limma’ package (v.3.58.1). In these datasets, 876, 632, and 340 DEGs were identified respectively. To find the targeted gene, a cross-analysis of the three DEGs sets was conducted. As shown in Fig. 1A, the intersection of these sets revealed 3 identical upregulated genes, yet no identical downregulated genes. LAMB1 was found to be associated with the development of several tumors in previous studies. However, LAMB1’s role and molecular mechanisms in NPC are yet to be elucidated. Consequently, we chose LAMB1 as a critical gene for subsequent research.

Western blot analysis confirmed elevated LAMB1 expression in NPC cell lines. Results indicated a significantly elevated LAMB1 expression in NPC cell lines CNE1 and CNE2 compared to the normal nasopharyngeal epithelial cell line NP69 (Fig. 1B). Furthermore, the association between LAMB1 and clinical stage, as well as TILs, were analyzed. Results demonstrated a correlation between high LAMB1 expression and advanced clinical stages (I vs. III, *p* = 0.01; I vs. IV, *p* = 0.01) (Fig. 1C), reduced intratumoral TILs (*p* = 0.03) (Fig. 1D). No significant difference was observed in stromal TILs (Fig. 1E). The single-cell map of NPC revealed that LAMB1 was expressed not only in malignant cells but also in stromal cells (Supplement Fig. S1). We assessed relative abundance of CAFs in NPC using 3 distinct methods. Results indicated increased CAFs in the group of high-expression LAMB1 (*p* < 0.001) (Fig. 1F‒H).

### Survival analysis depending on the expression of LAMB1

**Fig. 2:**
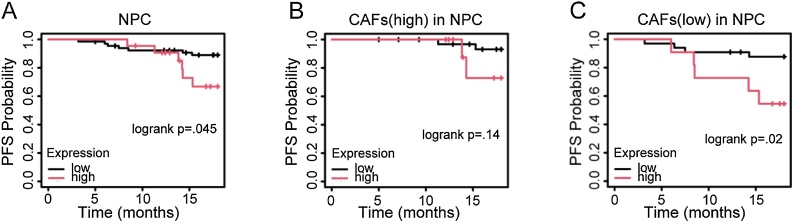
The survival analysis depending on the expression of LAMB1 in NPC. (A) PFS probability in NPC. (B‒C) PFS probability in high-CAFs and low-CAFs groups in NPC.

We conducted survival analyses in NPC. High-expression LAMB1 was associated with a poorer PFS probability (*p* = 0.045) (Fig. 2A). In low-CAFs group, LAMB1’s influence was more obvious (*p* = 0.02), but the difference was not significant in high-CAFs group (*p* = 0.14) (Fig. 2B‒C). These findings implied that LAMB1 played an important role in progression of NPC, especially in low-CAFs group.

### Functional enrichment analysis of LAMB1

**Fig. 3:**
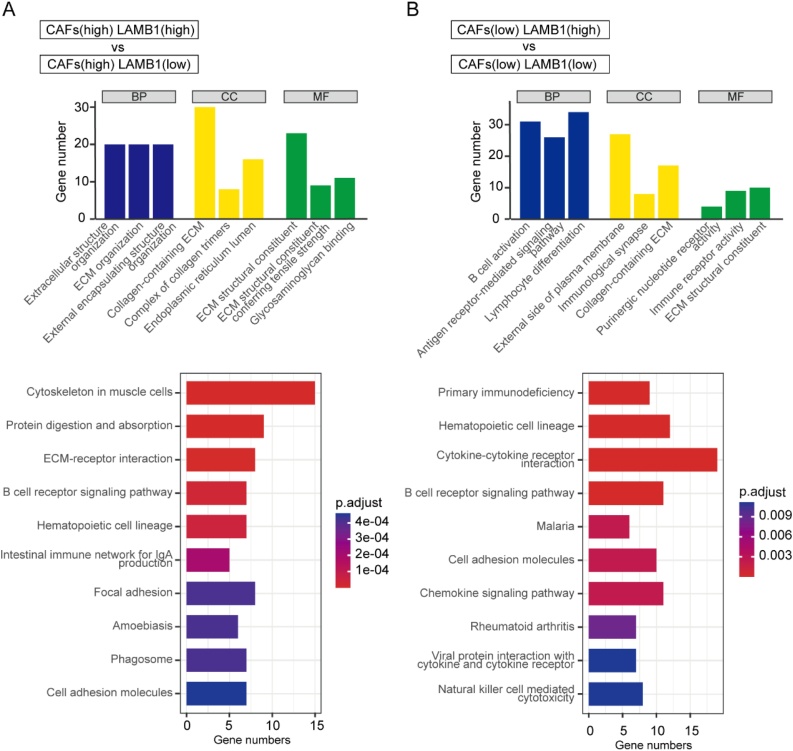
GO, KEGG analysis of DEGs in high/low expression of LAMB1 subsets of high/low-CAFs group. (A) The GO, KEGG analysis in high/low expression of LAMB1 subsets of high-CAFs group. (B) The GO, KEGG analysis in high/low expression of LAMB1 subsets of low-CAFs group.

The biological role of LAMB1 in NPC was explored through GO and KEGG analysis of DEGs in high-CAFs and low-CAFs groups separately. The results demonstrated that DEGs in the high-CAFs group were primarily enriched in ECM-related pathways (Fig. 3A), whereas in the low-CAFs group, enrichment predominantly concerned immune-related pathways (Fig. 3B). Additionally, high expression of LAMB1 was associated with cell adhesion related signaling pathways in both high and low CAFs groups.

### Correlation between LAMB1 and infiltrating immune cells in NPC

**Fig. 4:**
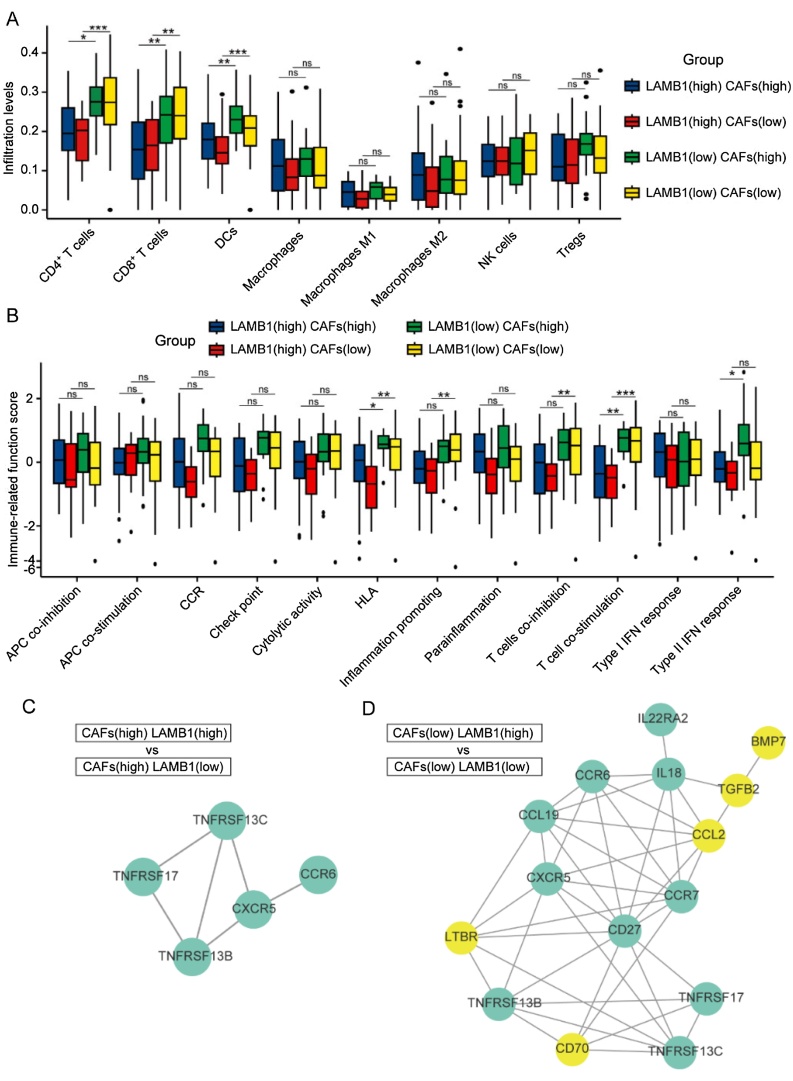
The analysis of immune cell infiltration, immune-related function score and differentially expressed cytokines in NPC. (A) The levels of 8 immune cells infiltration in high/low expression of LAMB1 subsets of high/low CAFs group. (B) The immune-related function score in high/low expression of LAMB1 subsets of high/low CAFs group. The differentially expressed cytokines between high and low expression of LAMB1 in the high (C) or low (D) CAFs groups. *p < 0.05, **p < 0.01, ***p < 0.001.

The correlation between LAMB1 expression and immune cell infiltration within TME was assessed using the xCell algorithm. The results demonstrated that in both high and low CAFs groups, high LAMB1 expression was associated with reduced infiltration of CD4^+^ T-cells, CD8^+^ T-cells and Dendritic Cells (DCs) compared to low LAMB1 expression (Fig. 4A). Subsequently, we analyzed the score of immune related function and differentially expressed cytokines in high-CAFs and low-CAFs groups separately. In low-CAFs group, high LAMB1 expression had lower score in HLA, inflammation promoting, T-cell co-inhibition and T-cell co-stimulation. While in high-CAFs group, LAMB1 high expression had lower score in HLA, T-cell co-stimulation and type II IFN response. And the difference in HLA and T-cell co-stimulation was more significant in low-CAFs group (Fig. 4B). Furthermore, the low-CAFs group exhibited a greater diversity of differentially expressed cytokines compared to the high-CAFs group (Fig. 4C‒D). These results indicated that high LAMB1 expression can inhibit the infiltration of T-cells, DCs, and B cells in NPC, but the mechanisms involved were different across varying CAFs levels.

### LAMB1 suppress HLA-1 and enhances the proliferation, migration and invasion of NPC cells

**Fig. 5:**
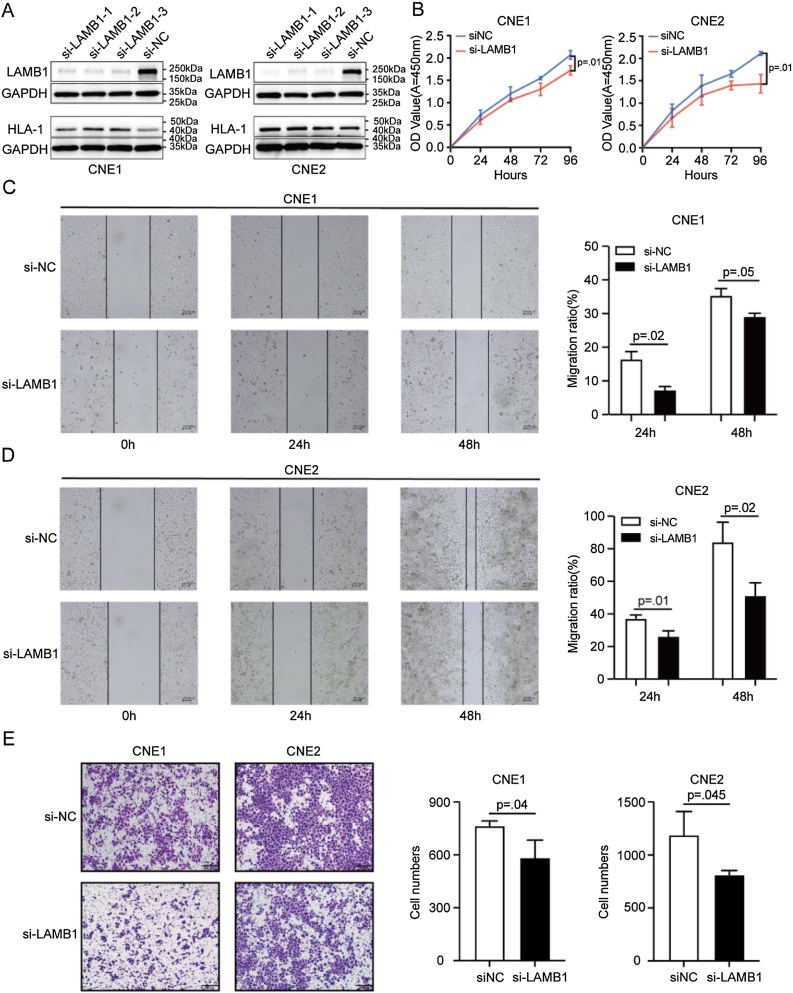
LAMB1 suppress HLA-1 and enhances the proliferation, migration and invasion of NPC cells. (A) Western Blot showed that the expression of LAMB1 was significantly decreased and the expression of HLA-1 was significantly increased in CNE1 and CNE2 after transfection. CCK8, wound scratch assay, and Transwell invasion assay showed that downregulation of LAMB1 can inhibit proliferation (B), migration (C‒D) and invasive abilities (E).

To explore LAMB1’s biological function in NPC cells, LAMB1 was knocked down in CNE1 and CNE2 cells. The effect of siRNAs has been validated by qPCR (Supplementary Fig. S2). Western blot analysis revealed a significant decrease of LAMB1 expression in transfected NPC cells (Fig. 5A). The expression of HLA-1 was increased after knocking down LAMB1 (Fig. 5A). The effects of LAMB1 on NPC cells were examined using CCK-8, wound scratch assay, and Transwell invasion assay. Results indicated that inhibition of LAMB1 expression significantly suppressed both proliferation (Fig. 5B) and invasive abilities (Fig. 5E). Moreover, there was a significant reduction in migration distance (Figs. 5C‒D). These findings indicated that LAMB1 downregulation can promote HLA-1 and impede NPC cells’ proliferation, invasion, and migration abilities.

### Prediction of immunotherapy response based on the expression of LAMB1

**Fig. 6:**
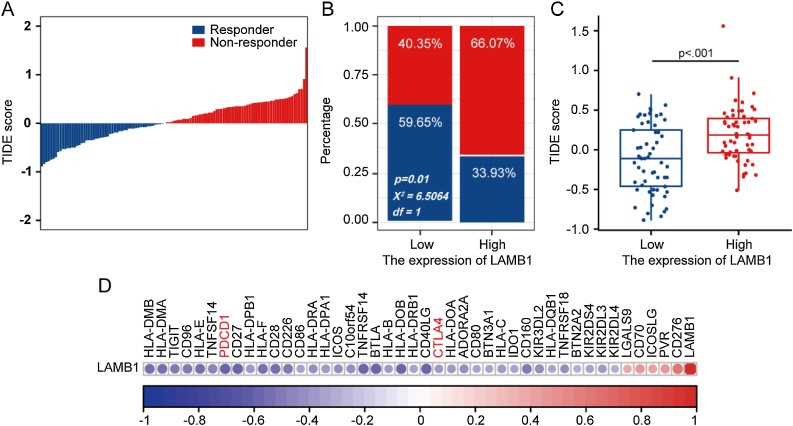
The analysis of TIDE and immune checkpoints. (A) The overall predicted TIDE score distribution. (B) The comparison of percentage of responder in high and low LAMB1 expression groups. The red represented non-responder, and the blue represented responder. (C) The TIDE score in different LAMB1 expression groups. (D) The association between immune checkpoints and LAMB1.

We used the TIDE module to predict the immunotherapy response in patients with different LAMB1 expression levels. The overall TIDE score distribution was depicted in Fig. 6A. Patients with low LAMB1 expression demonstrated lower TIDE scores, indicating a better immunotherapy response (Fig. 6B‒C). LAMB1 exhibited a negative correlation with the expression of most immune checkpoints, including PD-1 and CTLA-4 (Fig. 6D).

### Prediction of drugs sensitivity based on the expression of LAMB1

**Fig. 7:**
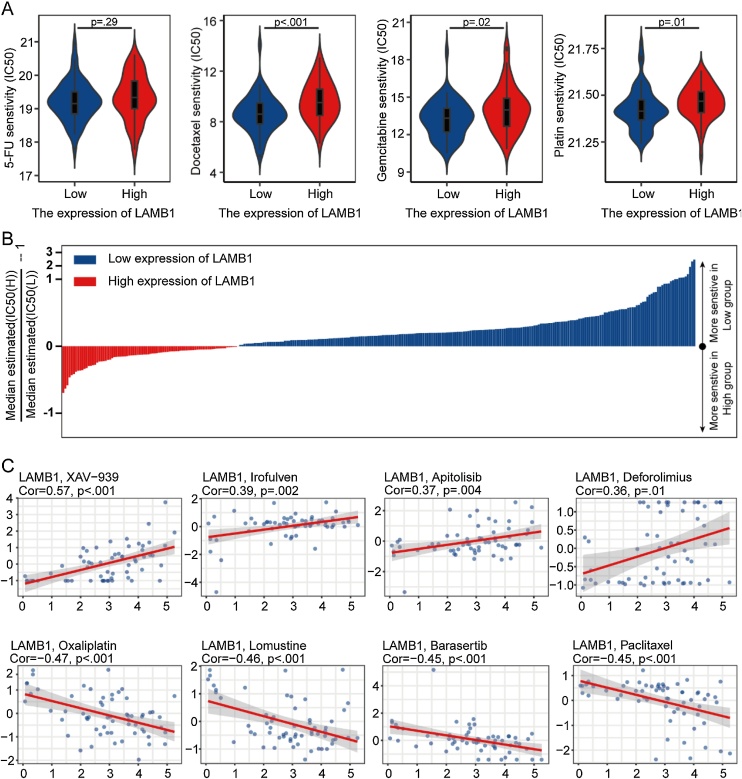
The prediction of drugs based on LAMB1 expression. (A) The predicted IC50 of platin, 5-FU, gemcitabine, and docetaxel in the GSE102349. (B) The normalized IC50 values of 300 compounds in the GSE102349. (C) The top 8 drugs positively and negatively associated with LAMB1 predicted by CellMiner database.

The CTRP database was used for the IC50 prediction of drugs in the GSE102349 dataset, encompassing 546 compounds in the analysis. Comparisons were conducted between LAMB1 high and low expression groups. Fig. 7B presented 300 compounds with *p* < 0.05 and normalized IC50 values, demonstrating increased drug sensitivity in the LAMB1 low expression group. Standard chemotherapeutic agents for NPC, including platin, 5-Fluorouracil (5-FU), gemcitabine, and docetaxel, were analyzed. The LAMB1 low expression group exhibited greater sensitivity to gemcitabine, platin and docetaxel, with no significant sensitivity difference observed for 5-FU (Fig. 7A). Subsequently, drugs associated with LAMB1 were predicted using the CellMiner database. Drug sensitivity was measured using Z-score, where higher scores indicated high drug sensitivity. The top four drugs positively associated with LAMB1 included XAV-939, Irofulven, Apitolisib, and Deforolimus, and those negatively associated comprised Oxaliplatin, Lomustine, Barasertib, and Paclitaxel (Fig. 7C).

## Discussion

The integration of radiotherapy, neoadjuvant chemotherapy, and immunotherapy has significantly improved the disease control rate among NPC patients. However, a particular subset of NPC patients still experiences distant metastasis after treatment. Further research into metastatic and recurrent NPC is imperative to uncover potential therapeutic targets. Upon analyzing three NPC datasets sourced from GEO database, we concentrated on the key differential expression gene, LAMB1. In this study, we observed that high LAMB1 expression was correlated with advanced stages and lower levels of TILs in NPC. Except malignant cells, we also observed a close relation between LAMB1 and CAFs. Consequently, we analyzed LAMB1’s function in high and low level of CAFs. Patients with high LAMB1 expression were more likely to experience progression in low CAFs group, but the difference was not significant in high CAFs group. These results suggest that LAMB1 originating from NPC cells may have a more potent effect on the progression of NPC than from other sources.

We also found LAMB1’s immunosuppressive function in TME. High LAMB1 expression was associated with reduced infiltration of CD4^+^ T-cells, CD8^+^ T-cells, and DCs in both high and low CAFs groups. Further investigation revealed that LAMB1 suppressed the expression of HLA molecules and the stimulation of T-cells. In-vitro experiment validated increasing expression of HLA-1 when knocking down LAMB1 in NPC cells. HLA-1 molecules are crucial for anticancer immunity. During the development of cancer, lots of neo-antigens are produced due to accumulated mutations. These neo-antigens are presented by HLA-1 molecules and recognized by T-cells, ultimately leading to the destruction of tumor cells.^9^ However, tumors employ various strategies to evade immune recognition, including the loss of HLA-1.^10^ Ogino et al. found that HLA-1 was downregulated in over 65% of NPC samples, and the infiltration of CD8^+^ T-cells was significantly correlated with the expression of HLA-1 (*r* = 0.34),^11^ suggesting that defect of HLA-1 is a significant mechanism of immune evasion in NPC. We identified a novel mechanism that the downregulation of LAMB1 could restore HLA-1 expression. Inhibition of LAMB1 may enhance the antitumor T-cell response and improve the efficacy of immunotherapy. Additionally, in high-CAFs group, only 5 cytokines showed differential expression, and the DEGs were primarily enriched in ECM-related pathways. In low-CAFs group, 15 cytokines showed differential expression, and the DEGs were predominantly enriched in immune-related pathways. As depicted in Fig. 4D, TGF-β2 and CCL2 were upregulated alongside LAMB1, while CCL19-CCR7 were downregulated alongside LAMB1. These 4 cytokines serve as crucial immune regulators. TGF-β can induce apoptosis in many cells, thereby inhibiting the release of new antigens.^12^ It also promotes abnormal tumor angiogenesis, leading to increased intratumoral pressure and consequently reduced T-cell infiltration.^13^, ^14^ Tumor cells can secrete CCL2, which recruits immunosuppressive cells, including myeloid-derived suppressor cells and Tregs.^15^ In prostate cancer, CCL2 has been shown to be involved in the formation of a metastatic niche.^16^ Moreover, one of the main functions of CCL19-CCR7 is to guide DCs chemotaxis.^17^ In turn, the downregulation of these two chemokines reduced the infiltration of DCs. In summary, LAMB1 influenced immune cells infiltration in NPC, and the mechanisms involved may differ depending on the CAFs levels.

We also predicted the impact of LAMB1 on the efficacy of NPC treatment. The TIDE score was significantly higher in the high LAMB1 expression group, indicating a greater likelihood of immune escape and a reduced response to Immune Checkpoint Inhibitor (ICI) therapy. Our analysis revealed a negative correlation between LAMB1 and immune checkpoint expressions, such as PD-1 and CTLA-4, potentially impacting the efficacy of immunotherapy. Furthermore, CAFs have the ability to regulate immune cell recruitment and activity through the secretion of cytokines and effectors, including TGFβ, CXCL2, collagen, and laminin.^5^ For example, TGF-β1 can stimulate tumor cells to express laminin γ2 via the JNK/AP1 signaling pathway, affecting T-cell receptor gene transcription and chemotaxis, contributing to ineffective anti-PD-1 therapy. Additionally, this study assessed LAMB1’s impact on standard chemotherapy for NPC. Platin, gemcitabine and docetaxel showed greater sensitivity in the low LAMB1 expression group. The findings suggested that TPF regimen (docetaxel + cisplatin + 5-FU) and GP regimen (gemcitabine + cisplatin) might offer more benefits to patients with low LAMB1 expression.

We also confirmed that LAMB1 promoted the proliferation, invasion, and migration of NPC cells. LAMB1, the β1 chain of laminin, has a C-terminus that binds to integrins, activating specific signaling networks that regulate cellular activities including adhesion, migration, and differentiation.^18^ Classical integrin outside-in signaling induces Focal Adhesion Kinase (FAK) phosphorylation, which activates the FAK/Src complex and subsequently leads to RHO GTPases-Cdc42 and Rac1 activation, resulting in rapid cell migration.^19^

In summary, our study observed that NPC exhibited significantly elevated expression of LAMB1, which was correlated with higher rate of progression and lower levels of TILs. Depending on CAFs levels, LAMB1 exerted an inhibitory effect on immune infiltration through different mechanisms. Immune checkpoints and HLA-1 were generally downregulated in NPCs with high LAMB1 expression. The molecular mechanisms underlying this phenomenon need further investigation. Subsequent experiments confirmed the critical role of LAMB1 in promoting proliferation, invasion and migration of NPC cells. This study has limitations: we utilized public databases to investigate the LAMB1 gene and its related functional mechanisms, which requires verification through additional in vitro and in vivo experiments.

## Conclusion

Our study revealed LAMB1’s pro-tumor role in NPC. High expression of LAMB1 promoted the progression of NPC by inhibiting immune responses and enhancing the malignant nature of tumor cells. In the future research, we will explore the impact of LAMB1 on immune escape in NPC, aiming to identify clinically viable targets for immunotherapy.

## CRediT authorship contribution statement

Xiaojiang Li and Enzi Feng contributed to the study conception and design. Material preparation, data analysis and data visualization were performed by Enzi Feng. Xingyu Yang performed the in vitro experiments. The first draft of the manuscript was written by Enzi Feng. Xiaojiang Li and Jie Yang reviewed the manuscript. Qianqian Qu helped prepare the discussion.

## Funding

This work was supported by the National Natural Science Foundation of China (Grant numbers 81960489) and the Research Foundation of Education Bureau of Yunnan Province, China (Grant numbers 2023Y0658).

## Declaration of competing interest

The authors declare no conflicts of interest.
