# Supplementary material for: LAMB1 promotes proliferation and metastasis in nasopharyngeal carcinoma and shapes the immune-suppressive tumor microenvironment
Source: Braz J Otorhinolaryngol. 2025 Jan 27;91(2):101551. doi: 10.1016/j.bjorl.2024.101551 (PMC11808599; doi:10.1016/j.bjorl.2024.101551)

**BJORL-D-24-00252_Supplementary Materials**

**Supplementary Materials Figure 1** LAMB1’s expression in the single-cell map of NPC.


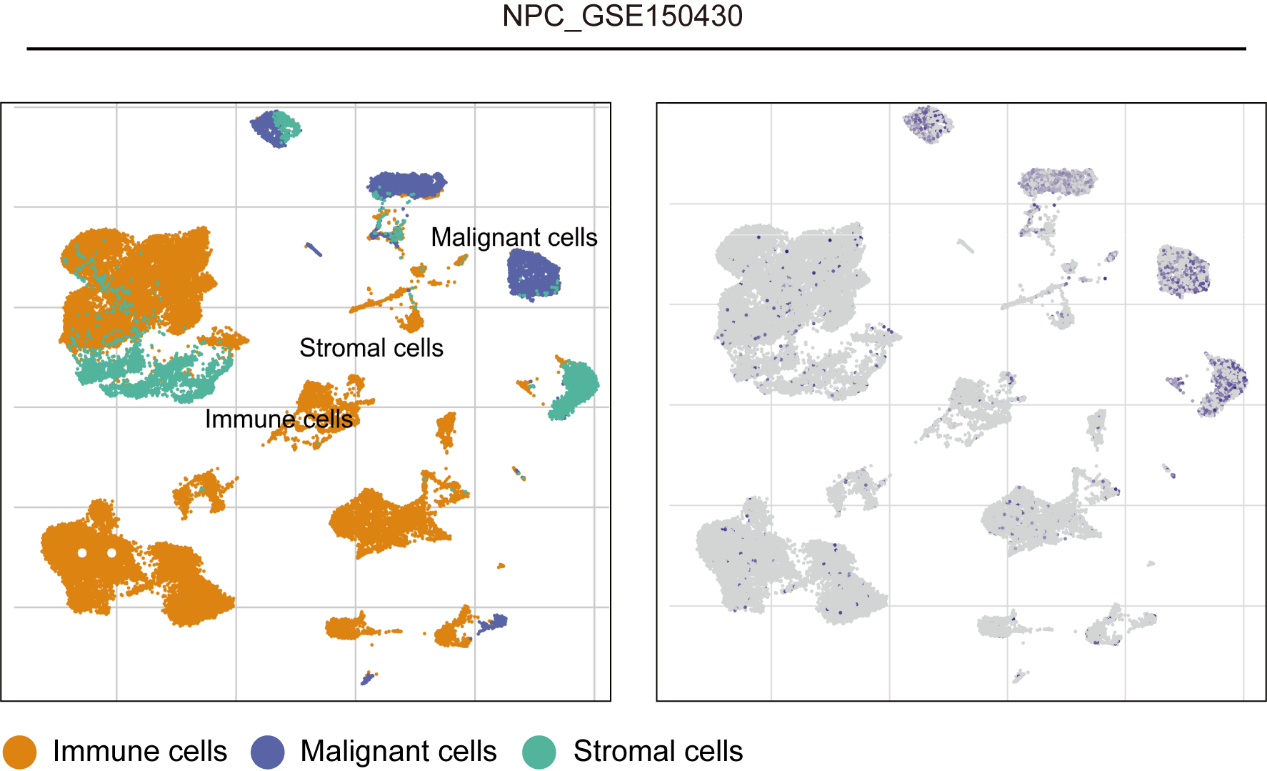


**Supplementary Materials Figure 2** The qPCR results of CNE1 and CNE2 transfected with siRNAs.


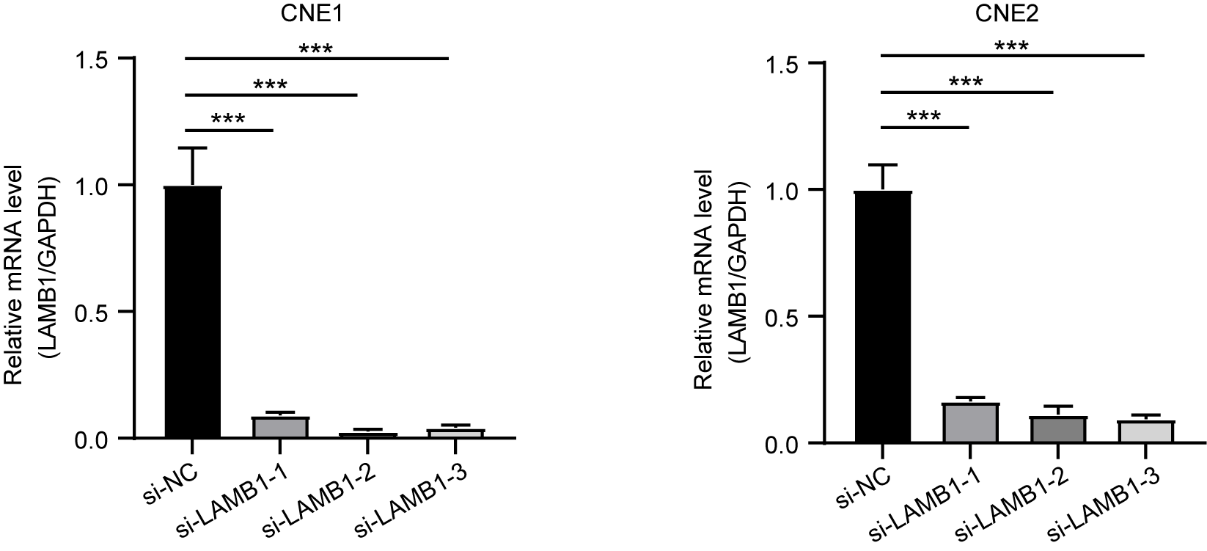

Supplement: Supplementary file 1 [file mmc1.docx]
